# Supplementary material for: Exploring the dynamic adult hard ticks-camel-pathogens interaction
Source: mSphere. 2024 Oct 29;9(11):e00405-24. doi: 10.1128/msphere.00405-24 (PMC11580451; doi:10.1128/msphere.00405-24)
Supplement: Supplemental material — Supplemental text, tables, and figures. [file msphere.00405-24-s0001.docx]

**Exploring the dynamic adult hard ticks-camel-pathogens interaction**

Supplementary Materials

**JohnMark O. Makwatta^1,2^, Paul N. Ndegwa^2^, Florence A. Oyieke^2^, Peter Ahuya^1^, Daniel K. Masiga^1^, Merid N. Getahun^1,^**

[**^mgetahun@icipe.org^**](mailto:mgetahun@icipe.org)

**Supplementary Results**

Morphological description of tick species

The hard tick species' sexes were determined by having a sclerotized plate that only covered the female’s anterior dorsal region but covered the entire male’s back which restricts the extension of the male body following a blood meal. *Amblyomma lepidum* females were morphologically identified by large conspicuous punctations. Male *A. lepidum* was distinguished by a partial enamel pattern with spots on 6 to 8 of the 11 festoons, and the absence of an enamel on the central and two outermost festoons.

The genital aperture of *Hyalomma dromedarii* ticks had a slight preatrial fold. The conscutum of male *H. dromedarii* had short lateral grooves and four posterior ridges. The males’ sub-anal plates were conspicuously large and distinctly aligned outside the adanal plates irrespective of feeding status.

Female *Hyalomma impeltatum* had a scutum with a distinct sinuous posterior margin in comparison with *H. dromedarii*’s slightly sinuous. The moderately convex preatrial fold on the genital aperture was depressed posteriorly but bulged anteriorly. A distinctive feature to this *Hyalomma* species was the slight bulge bordering each side of the genital aperture giving the genital area a trilobed appearance. Male *H. impeltatum* had a pale central festoon and a conscutum with two posterior ridges and long lateral grooves. Unfed male specimens had small sub-anal plates that were vertically aligned with the adanal plates.

*Amblyomma varietagum* males were characterized by having a patterned enamel without the lateral spots. Besides having convex eyes that were separate from the margin of the scutum, the enamel of both *A. variegatum* sexes was characteristically pink to orange in color with small to medium punctations that differentiated this tick species from *Amblyomma lepidum*.

*Hyalomma rufipes* was characterized by a robust, large, shiny black scutum. The punctations of both sexes were dense and covered the scutum and conscutum evenly. The integument area had dense setae surrounding the spiracles. Unlike the other *Hyalomma* species, *H. rufipes* male scutum was evenly-rounded and lacked depressions and grooves. The genital aperture had a deep anterior grove from which the preatrial fold strongly bulged.

Both *Hyalomma marginatum* sexes were identified by lack of both dense punctations and dense setae around the spiracles. The female preatrial fold was very complex. *H. marginatum* male was confirmed by a caudal depression, with posterior grooves and a brown colored central festoon.

In collections of *Hyalomma* ticks, the males of *H. truncatum* species stood out by a shiny, smooth and dark conscutum exemplified by a large, depressed area (concave) in the caudal (posterior) area. The dense punctations on the depressed caudal area gave the ticks a contrasting rough appearance. The preatrial fold of female *H. truncatum* was concave.

Coxa 1 of *Rhipicephalus appendiculatus* males had a prominent anterior spur visible from the dorsal side. Unlike all the identified species, the males of *R. appendiculatus* were characterized by sharply raised margins on the broad cervical fields with slightly convex eyes, fewer punctations, and not deeply sunken posterior grooves. The female *R. appendiculatus* were differentially diagnosed with having the same characteristics as their counterpart males.

Female *Rhipicephalus camicasi* were distinctly identified by their shallow scapular grooves, and narrow tails on the spiracles plates. The spiracle plate tails were half the width of the adjacent festoon. Female *R. camicasi* were collected only from camels.

*Rhipicephalus pulchellus* was characterized by white on black stripes with males distinguished by a dark brown background over which there were patterns of white enamel stripes covering the entire conscutum.

Both sexes of *Rhipicephalus pravus* were confirmed by morphological identification of the straight scapular grooves that defined their small cervical fields. The punctations were dense interstitial and distinct setiferous respectively. This tick species was identified from camels, goats and sheep.

*Rhipicephalus sanguineus* females were diagnostically identified by their broad genital aperture. Both sexes had spiracle plates with narrow tails. The tails were less than the width of the adjacent festoon. The males did not have depressions of the cervical fields. The identified *R. sanguineus* ticks were from camels.

The male *Rhipicephalus praetextatus* was identified among ticks collected from camels and was characterized by dense distribution of small interstitial punctations.

All the identified ticks collected from camels and other livestock were adults. The unresolved *Amblyomma*, *Hyalomma* and *Rhipicephalus* species were mainly engorged females or males with distorted identification features.

Table S1: Primers for DNA amplification

| **Primer** | **Target gene** | **Primer sequence (5’ – 3’)** | **Product size (bp)** |
| --- | --- | --- | --- |
| TITS2 F  TITS2 R | ITS2 | CGAGACTTGGTGTGAATTGCA  TCCCATACACCACATTTCCCG | 920 – 1850 |
| 16S + 1  16S – 1 | 16S rRNA | CTGCTCAATGATTTTTTAAATTGCTGTGG  CCGGTCTGAACTCAGATCAAGT | 460 |
| SR-J-14199F  SR-N-14594R | 12S rRNA | TACTATGTTACGACTTAT  AAACTAGGATTAGATACCC | 430 |
| LCO1490  HCO2198 | CO1 | GGTCAACAAATCATAAAGATATTGG  TAAACTTCAGGGTGACCAAAAAATCA | 710 |
| EHR 16SD  pH1492 | *Anaplasma*/*Ehrlichia* 16S rRNA | GGTACCYACAGAAGAAGTCC  GGTTACCTTGTTACGACTT | 1030 |
| EHRF  EHRR | *Ehrlichia* 16S rRNA | AGCTGGTCTGAGAGGACGAT  GAGTGCCCAGCATTACCTGT | 838 |
| ompB 120-2788  ompB 120-3599 | *Rickettsia ompB* | AAACAATAATCAAGGTACTGT  TACTTCCGGTTACAGCAAAGT | 836 |
| Trans1  Trans2 | *Coxiella* IS1111 | TGGTATTCTTGCCGATGAC  GATCGTAACTGCTTAATAAACCG | 687 |
| RLB F  RLB R | *Theileria*/*Babesia* 18S rRNA | GAGGTAGTGACAAGAAATAACAATA  TCTTCGATCCCCTAACTTTC | 500 |
| ITS1 – CF  ITS1 – BR | *Trypanosoma* sp. | CCGGAAGTTCACCGATATTG  TTGCTGCGTTCTTCAACGAA | 250 – 710 |

Table S2: Thermocycling conditions for genes amplified in the Proflex PCR system.

The initial denaturation for all the amplifications were set at 95°C for 15 minutes, preceding the conditions in the table above. The thermal cycler was set with an infinite hold at 8°C upon completion of the amplification before analyses by agarose gel electrophoresis.

| **Target gene** |  |
| --- | --- |
| ITS2 | 35 cycles of denaturation at 95°C for 30 s, annealing at 65°C for 30 s, 72°C extension for 1 min; and 1 cycle for final extension at 72°C for 10 min |
| 16S rRNA | first 15 cycles split into 5 cycles each with the same initial denaturation at 94°C but different annealing temperatures of 49°C, 47°C and 45°C, and extension at 72°C followed by subsequent 25 cycles of initial denaturation at 94°C, annealing at 43°C, extension at 72°C and 1 cycle final extension at 72°C for 5 min – all the preceding steps were set at 30 s |
| 12S rRNA | 35 cycles of denaturation at 95°C for 30 s, annealing at 48°C for 30 s (65°C for ITS2), 72°C extension for 1 min; and 1 cycle for final extension at 72°C for 10 min |
| CO1 | 40 cycles – (95°C for 45 s, annealing at 53°C for 1 min, initial extension at 72°C for 1 min); and 1 cycle for final extension at 72°C for 5 min |
| *Anaplasma/Ehrlichia 16S rRNA* | 3 cycles - 95°C for 20 sec, 61°C for 30 sec, 72°C for 1 min 30 sec followed by 37 cycles of 95°C for 30 sec, 60°C for 30 sec, and 72°C for 1 min 20 sec; final extension at 72°C for 10 minutes |
| *Ehrlichia* 16S rRNA | 35 cycles - 95°C for 45 sec, 53°C for 30 sec, and 72°C for 40 sec; final extension 72°C for 7 minutes |
| *Rickettsia ompB* | 35 cycles - 94°C for 30 sec, 62°C for 45 sec, and 72°C for 45 sec; final extension 72°C for 7 minutes |
| *Coxiella* IS1111 | 5 cycles - 95°C for 30 sec, 66°C-61°C for 45 sec (touchdown), 72°C for 45 sec followed by 35 cycles of 95°C for 30 sec, 61°C for 30 sec, and 72°C for 45 sec; final extension at 72°C for 7 minutes |
| *Theileria/Babesia 18S rRNA* | 5 cycles - 95°C for 30 sec, 60°C-55°C for 30 sec (touchdown), 72°C for 1 min followed by 30 cycles of 95°C for 30 sec, 55°C for 30 sec, and 72°C for 1 min; final extension at 72°C for 10 minutes |
| *Trypanosoma spp.* | 35 cycles- 95°C for 20 sec, 62°C for 30 sec, 72°C for 30 sec; final extension 72°C for 7 min |

Table S3: Accession numbers for the ticks identified using molecular markers and their percent identity matches by BLASTn for reference sequences in GenBank.

| Tick species | Accession Number(s) | | | |
| --- | --- | --- | --- | --- |
|  | 12S rDNA | 16S rDNA | ITS2 rDNA | CO1 |
| *A.gemma* | OQ565131, 133 | OQ566200, 202 | OQ557777, OQ557781, OQ557783 | OQ540938, OQ540940 |
| *A.lepidum* | OQ565134, OQ565136, OQ565143 | OQ566203, OQ566204 | OQ557775 (New) | OQ540941, OQ540943 |
| *A.variegatum* | OQ565138, OQ565139, OQ565144 | OQ266206, OQ566207 | OQ557778, OQ557779 | OQ540945 - OQ540946 |
| *H.rufipes* | OQ565126,  OQ565137, OQ565147, OQ565148 | OQ566194, OQ566195, OQ566215, OQ566216 | - | OQ540935, OQ540936, OQ540949 |
| *H.dromedarii* | OQ565122 – OQ565133 | OQ566191, OQ566192 | OQ557790, OQ557792 – OQ557794 | OQ540932 |
| *H.impeltatum* | OQ565124, OQ565125 | OQ566193 | OQ557773, OQ557774, OQ557785 (New) | OQ540933, OQ540934 |
| *H.truncatum* | - | OQ566205 | OQ557780 | OQ540944 |
| *H.marginatum* | OQ565140 | OQ566208 | - | OQ540947 |
| *R.appendiculatus* | OQ565129, OQ565130 | OQ566198, OQ566199 | OQ557772, OQ557788 | OQ540937 |
| *R.camicasi* | OQ565142, OQ565146 | OQ566214 | - | - |
| *R.pravus* | OQ565120, OQ565121 | OQ566189, OQ566190 | OQ557778, OQ557779, OQ557782 (New) | - |
| *R.pulchellus* | OQ565127, OQ565128 | OQ566196, OQ566197 | OQ557784 | - |
| *R.sanguineus* | - | OQ566209, OQ566210 | - | - |
| *R.praetextatus* | OQ565145 | OQ566213 | - | - |

Table S4**:** Diversity and abundance of ticks collected from camels and co-herding livestock between 2019 and 2022

| **Tick species** | **Male** | **Female** | **Total** | **Abundance (%)** |
| --- | --- | --- | --- | --- |
| *H.rufipes* | 616 | 262 | 838 | 27.7 |
| *H.dromedarii* | 532 | 247 | 779 | 25.8 |
| *H.impeltatum* | 208 | 103 | 311 | 10.3 |
| *A.gemma* | 165 | 90 | 255 | 8.4 |
| *R.pulchellus* | 86 | 74 | 160 | 5.3 |
| *R.pravus* | 77 | 63 | 140 | 4.6 |
| *Hyalomma_Spp.* | 11 | 118 | 129 | 4.3 |
| *Rhipicephalus_Spp.* | 0 | 114 | 114 | 3.8 |
| *H.marginatum* | 73 | 32 | 105 | 3.5 |
| *H.truncatum* | 55 | 33 | 88 | 2.9 |
| *R.sanguineus* | 19 | 9 | 28 | 0.9 |
| *A.lepidum* | 14 | 13 | 27 | 0.9 |
| *R.appendiculatus* | 9 | 12 | 21 | 0.7 |
| *A.variegatum* | 5 | 13 | 18 | 0.6 |
| *Amblyomma_Spp.* | 0 | 5 | 5 | 0.2 |
| *R.camicasi* | 0 | 2 | 2 | 0.07 |
| *R.praetextatus* | 1 | 0 | 1 | 0.03 |

Table S5: Tick counts on individual camel predilection sites

| **Camel code** | **Eye** | **Ear** | **Nose** | **Body trunk** | **Belly** | **Udder** | **Tail** | **Anal region** |
| --- | --- | --- | --- | --- | --- | --- | --- | --- |
| Camel 1 | 0 | 0 | 15 | 0 | 1 | 1 | 18 | 10 |
| Camel 2 | 1 | 6 | 5 | 0 | 0 | 2 | 1 | 0 |
| Camel 3 | 0 | 0 | 0 | 1 | 8 | 0 | 2 | 11 |
| Camel 4 | 0 | 0 | 6 | 0 | 0 | 0 | 7 | 9 |
| Camel 5 | 1 | 0 | 7 | 0 | 0 | 1 | 0 | 0 |
| Camel 6 | 0 | 1 | 0 | 0 | 5 | 0 | 10 | 8 |
| Camel 7 | 2 | 5 | 16 | 0 | 6 | 0 | 0 | 0 |
| Camel 8 | 1 | 2 | 0 | 0 | 0 | 0 | 5 | 9 |
| Camel 9 | 0 | 0 | 0 | 0 | 0 | 1 | 0 | 14 |
| Camel 10 | 1 | 0 | 6 | 0 | 0 | 0 | 0 | 0 |
| Camel 11 | 0 | 0 | 0 | 0 | 0 | 0 | 7 | 0 |
| Camel 12 | 0 | 0 | 12 | 0 | 1 | 0 | 0 | 12 |
| Camel 13 | 1 | 2 | 0 | 0 | 0 | 0 | 14 | 0 |
| Camel 14 | 0 | 0 | 0 | 0 | 0 | 0 | 0 | 5 |
| Camel 15 | 0 | 0 | 8 | 0 | 0 | 0 | 2 | 0 |
| Camel 16 | 0 | 1 | 0 | 0 | 0 | 1 | 0 | 0 |
| Camel 17 | 0 | 0 | 7 | 0 | 0 | 0 | 3 | 0 |
| Camel 18 | 0 | 0 | 30 | 0 | 3 | 0 | 8 | 16 |
| Camel 19 | 0 | 0 | 0 | 0 | 0 | 0 | 6 | 0 |
| Camel 20 | 1 | 0 | 10 | 0 | 0 | 0 | 0 | 0 |
| Camel 21 | 0 | 0 | 0 | 0 | 0 | 0 | 1 | 0 |
| Camel 22 | 0 | 0 | 13 | 0 | 0 | 0 | 0 | 0 |
| Camel 23 | 0 | 0 | 8 | 0 | 0 | 0 | 8 | 0 |
| Camel 24 | 0 | 0 | 0 | 0 | 4 | 0 | 0 | 0 |
| Camel 25 | 0 | 1 | 14 | 0 | 0 | 0 | 0 | 1 |
| Camel 26 | 0 | 0 | 0 | 0 | 3 | 0 | 1 | 0 |
| Camel 27 | 0 | 0 | 5 | 0 | 0 | 0 | 0 | 0 |
| Camel 28 | 0 | 0 | 11 | 0 | 0 | 0 | 0 | 0 |
| Camel 29 | 0 | 0 | 0 | 0 | 0 | 1 | 1 | 0 |
| Camel 30 | 0 | 0 | 0 | 0 | 5 | 0 | 0 | 0 |

Table S6: Cumulative Tick species count per predilection sites on camel

| **Tick species** | **Eye** | **Ear** | **Nose** | **Body trunk** | **Belly** | **Udder** | **Tail** | **Anal region** |
| --- | --- | --- | --- | --- | --- | --- | --- | --- |
| *H. rufipes* | 2 | 2 | 10 | 1 | 0 | 0 | 26 | 79 |
| *H. impeltatum* | 1 | 0 | 4 | 0 | 0 | 2 | 1 | 0 |
| *H. truncatum* | 0 | 0 | 2 | 0 | 0 | 0 | 10 | 0 |
| *H. dromedarii* | 0 | 16 | 131 | 0 | 21 | 0 | 40 | 5 |
| *R. camicasi* | 0 | 0 | 1 | 0 | 0 | 0 | 0 | 0 |
| *R. pravus* | 5 | 0 | 19 | 0 | 0 | 0 | 8 | 0 |
| *R. sanguineus* | 0 | 0 | 2 | 0 | 0 | 0 | 0 | 0 |
| *R. pulchellus* | 0 | 0 | 0 | 0 | 0 | 0 | 1 | 1 |
| *Rhipicephalus* spp*.* | 0 | 0 | 0 | 0 | 3 | 0 | 0 | 2 |
| *A. gemma* | 0 | 0 | 4 | 0 | 12 | 5 | 0 | 8 |
| A. lepidum | 0 | 0 | 0 | 0 | 0 | 0 | 8 | 0 |


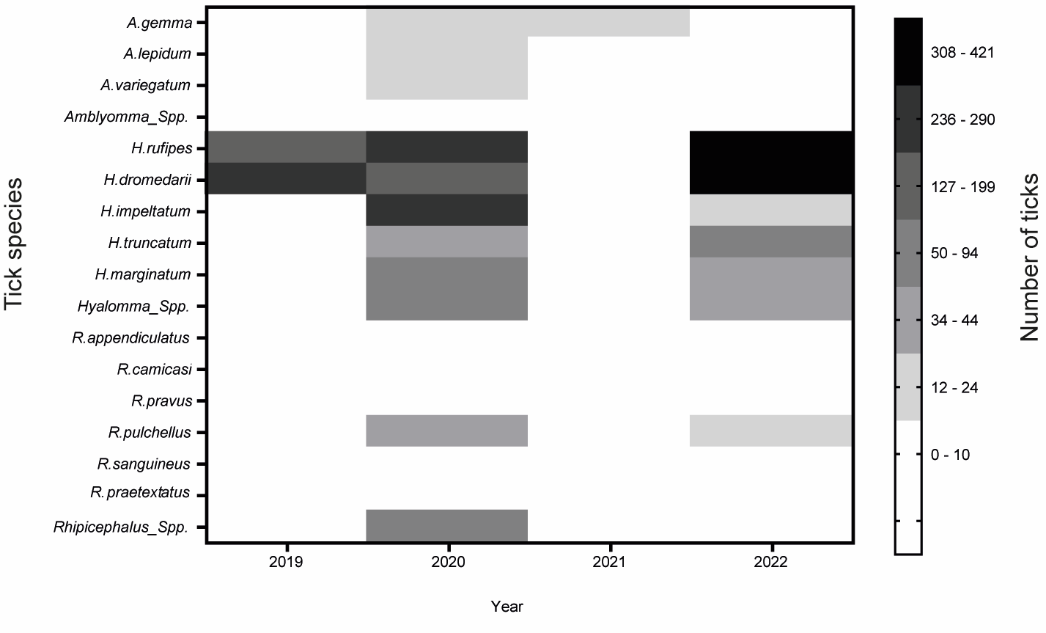


**Fig. S1.** Tick density on Camels from 2019 to 2022


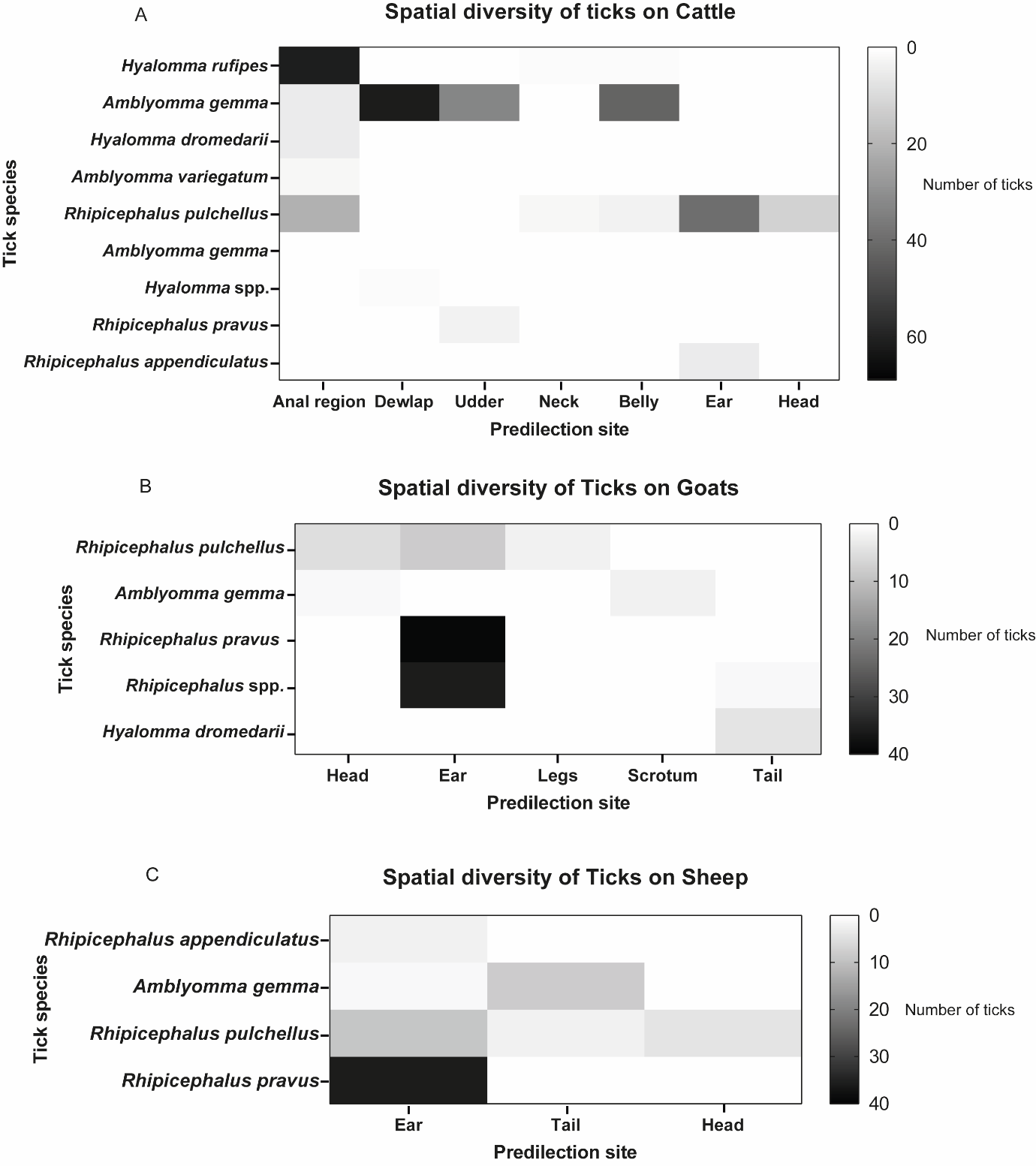


**Fig. S2.** Heatmap matrices of tick spatial diversity on predilection sites of co-herding livestock. Various ticks on different bodies of (A) Cattle (B) Goats and (C) Sheep.
